# Dr. Drake and His Critics

**Published:** 1854-07

**Authors:** 


### DR. DRAKE AND HIS CRITICS.

OUR friend, Dr. JOHN BARTLETT, has translated from the *Vierteljahrschrift fur die Praktische Heilkunde*, published at Prague, a part of a critical notice of Dr. DRAKE's work on the Principal Diseases of the great Interior Valley of North America, from which we make a few extracts.

"Here the first part of the work closes, and the author adds that all the diseases are treated of in direct reference to soil and climate, and that their consideration will immediately follow the first book on general Ætiology, accordingly as they most tend to illustrate the importance of the facts therein given. The author concludes with the modest remark, that much must be added to the book, that many mistakes must be corrected before a general reception can be demanded for it.

"He will, he says, never regret his labor, if he should have excited the attention of any young physician to an accurate investigation of fever; or if he prove instrumental in the recovery of a single patient, who otherwise would have fallen a victim to this disease. Unfortunately this decided tendency to the practical is often coupled with a neglect of a rigorous investigation of pathological questions. What renders this most objectionable is the neglected view of the symptomatic examination of diseases, the neglect of ontological, pathological and anatomical facts, as well as a great inattention to physical diagnosis, and to great dependence upon the subjective symptoms. Whence we conclude one is not always convinced by objective truths.

"We cannot be persuaded that all the cures reported as chronic hepatitis were really such, when he cites for us only pain, jaundiced color, hypochondriacal sensibility, &c., as symptoms—not one post-mortem confirms the assumed diagnosis. And the autopsies furnished by other cases are very imperfect; the examination is limited to an inspection of those organs which were expected to be found diseased. Nor do we find one instance of a thorough and accurate description of the condition of all the organs of the body. Besides, the number of post-mortems made were too insignificant to establish the many inferences drawn from them.

"The observations made are likewise imperfect and unsatisfactory, especially upon the physical examination of the chest. We can form absolutely no conception of the condition of the lungs, when the author says of a patient, "By percussion I found resonance loud and hollow, even over the spleen; his respiration was a little bronchial." No statement as to the condition of the secretions, as to reaction, as to their chemical retention, as to their quantity before and after a paroxysm of fever, before and after the exhibition of quinine, we find to be made. Yet such investigations as these would have been of great scientific interest, and on account of the great number of his cases of practical utility. It may be in this way only, that we can succeed in solving such questions as these, viz: by obtaining an accurate knowledge of the changes, the chemistry of the sick man undergoes, and thence determining the specific influence the alkaloid of Cinchona produces in the economy. Whether the author had time and opportunity for prosecuting such researches we do not know. It is not our aim to find fault, but only to point out here the way in which a discovery might have been given to science.

"As to the Therapeutics, they border close upon gross empiricism, and the indications set down by the author are often very widely removed from such as conduce to a rational practice,—nay, they are often as unintelligible as the modus operandi of his remedies. For instance, the author says of remittent fever, 'It may be the duty of the physician to convert the remittent into an intermittent, or to bring about skilfully an intermission, &c.'"

It is not very safe to judge of an author by a translation in another tongue, and we are not sure that the sense of the German critic here quoted is accurately given. If it is, we must say that much of his criticism appears to us extremely stupid. We have given the best portions of it. The critic labored under the disadvantage, probably, of not being familiar with the English language, and this explains some of the absurdities in his notice of Dr. Drake's work. We are not willing to believe that he was guilty of wilfully misrepresenting Dr. Drake's statements; as for example, when he says that "he cannot be persuaded that all the *cures reported* as chronic hepatitis were really such." Dr. Drake would be supposed from this language to have written fully of chronic hepatitis, and to have reported numerous cures; when the fact is, that he only alludes to the affection briefly, as one of the *sequelæ* of autumnal fever, and speaks of no cures. Not a case is given in detail. The critic says that as evidence of the existence of this condition of the liver, Dr. Drake mentiones "only pain, jaundiced color, hypochondriacal sensibility, &c." So far from this assertion being true, Dr. Drake gives the following long catalogue of rational symptoms:

The symptoms of subacute or chronic hepatitis, are constipation or diarrhœa; a suspended, depraved, or increased secretion of bile; acidity and irritability of stomach; variable appetite; in general, a foul and yellowish tongue; more or less jaundice of the skin and eyes, with yellowness of the urine; tenderness, and sometimes pain in the epigastric and right hypochondriac regions; aching about the right shoulder, sometimes descending into the arm; inconvenience in lying on the left side; a hacking cough, without expectoration; a dry, harsh, and insensible skin, with coldness of the feet; occasional flushes of fever, according to the degree of inflamation; almost constant frequency of the pulse, with fits of palpitation of the heart; reduced activity of mind, whimsicality, despondency, irresolution, and fear of death. In addition to the direct sympathy of various parts of the body with the liver, they sympathize with the stomach, which is dyspeptic; with the bowels, from which the liver withholds a due supply of bile, or irritates, with that which is unhealthy; but, above all, the whole nervous system, and, indeed, all the tissues of the body, are irritated by the bile, or its elements, which float with the circulating currents, and act on the exquisitely, susceptible, interior membrane of the arteries."

This reviewer complains that Dr. Drake's work contains few autopsies. No one regretted this defect more than the author himself; but it was unavoidable—the autopsies had not been made. He quotes the results of all that had been published by his American brethren.

The reviewer objects also to Dr. Drake's therapeutics, which he says "are little better than empirical," and sometimes "unintelligible." He cannot understand how a case of remittent may be converted into one of intermittent fever. American physicians generally think the thing may be done. They suppose that, by depleting and cooling medicines, the type of the fever is frequently changed, the remissions passing into intermissions, when quinine is administered and a cure speedily effected.

We think it doubtful, in the first place, whether the translation from which we have made the foregoing extracts does justice to the original; and in the next place, it is quite as doubtful whether the work of Dr. Drake was understood by the reviewer. We are sure there are many of his critical remarks which, if they contain any sense, cannot be understood by the English reader.

As an offset to these strictures of the critic of Prague, we give a few sentences from notices of Dr. Drake's book by physicians of London and Philadelphia.

The *British and Foreign Medico-Chirurgical Review* begins a notice thus:

"The above elaborate title belongs to as elaborate a work, the first volume of which only has yet appeared. The title expresses also clearly enough the design and purport of the treatise, which has no less an object than a complete medical history of the interior valley of North America. This history comprises, therefore, full details of the topography of the whole of this immense district, the condition of its inhabitants, and the mode in which they are affected by the climatic and social influences which surround them. The labor which is necessary for making a complete medical history of a single place is very great. The original geological conformation of the soil, the geographical relations, and the connected atmospheric conditions of the country,—the changes which bave been produced in it by cultivation,—the races which dwell on it, their mode of life, their political and social state, which may be consequent either on the influences of their own territory, or on the inherent impulses of race, or on the impressions derived from the surrounding states,—in fact, physical and moral influences, of whatever kind, are included in this term of complete medical history."

And after thirty pages of analysis and favorable remark, the reviewer closes with the following sentiment:

"We sincerely hope, however, that there is no danger of so useful a work not meeting with sufficient encouragement in America, to induce its veteran author to complete it without delay. We have had occasion to observe of late upon the dearth of medical works in that country, having pretensions to originality and research; native talent seeming to be wholly expended in dishing up and garnishing the productions of European pens; and it will indeed be melancholy to find an author, who has had the courage to produce a work of this magnitude, and one which would do honor to any country, left the sole consolation of the hope of a posthumous reputation."

Here is a notice of Dr. Drake's book, by a man with American feelings, and of a mind capacious enough to take in the subject. It is from the pen of that fine scholar and true physician, Prof. Stillé, and makes a part of his *Report on Medical Literature* read in 1850, to the American Medical Association:

"Of the original works in this catalogue, the most extensive and important is the first volume of Dr. Drake's *Treatise on the Principal Diseases of the Interior Valley of North America.* This volume, which contains nearly nine hundred pages, is taken up with the medical topography, the climate, the manners of the inhabitants, and the autumnal fevers of this vast region. Owing to the late period of its publication, the Committee have been unable to examine the treatise thoroughly, and they might, therefore, rest contented with a bare announcement of its existence. But they are reluctant to do so, since even a superficial inspection of the work has convinced them that it belongs to the very highest rank of our medical literature, and may very probably come to be regarded as the most valuable original work yet published in America. It is certainly unrivalled in the amount and variety of its materials; its style is perspicuous, correct, and elevated; and it appears to have been elaborated with great industry and care. Its distinguished author has raised a durable monument to his own name, and to the medical reputation, not only of the Great Valley, but of the greater Union."

This is a work of which a Louisville critic has lately spoken, as the production of "a babbling historiographer of disease, who had spent most of his life in running about the country collecting its medical gossipry." To a genial critic of our National Association, it appeared a work that did honor to the "great Union;" and to a just English reviewer, it seemed one which "would do honor to *any* country." But this cynical reviewer at home, who fancies that he was injured by Dr. Drake many years ago, can see nothing in the great work but "medical *gossipry*." The immense labor of traveling over our vast valley, observing its medical topography, and conferring with its intelligent medical men, was nothing in the jaundiced eyes of this small critic but "*gossipry.*"

Modesty, or, in the absence of higher motives, considerations of policy, one would think, might have restrained this writer from the utterance of such expressions concerning a book of which the press and the profession have everywhere agreed in saying, that nothing more honorable to American medical literature has yet appeared. A sentiment of patriotism, it might have been hoped, would have made him forget the petty bickerings of half a lifetime now that the grave had closed over the mortal remains of his fancied enemy. But no such feelings seem to have disturbed his settled malice, and in giving vent to his spleen he appears not to have considered that he might offend that large and respectable body of physicians whose experience in disease forms the staple of Dr. Drake's treatise. In sneering at the oral communications of these pains-taking, observant practitioners as "medical gossipry,"

it is strange he could not see that his censure fell more upon them than upon the philosophical traveler who was inquiring of their experience. "Babbling historiographer of disease!" "Medical gossipry!" These are the terms in which a mortified Louisville critic gives expression to his private griefs. They are as applicable to one party as to the other. They describe just as truly the gifted author of the work, as they do the intelligent men whose observations it records. If the Cartrights, the Fenners, the Dowlers, the Fearns, the Bolings, the Jennings, the Dawsons, the Carrolls, the Suttons, the Clapps—the thousand hard-working men in the profession, South and West—are "medical gossips," then, indeed, was Drake "a babbling historiographer of disease."

