# A Correction

**Published:** 1854-08

**Authors:** 


### A CORRECTION.

We have received the following note from Dr. Bartlett:—

Dr. Yandell:

*Dear Sir;* I have just read your notice of the critical remarks of Dr. Tinger upon Dr. Drake's work.

The word *cures* in the sentence upon which you have properly enough commented, is but one of the many and gross typographical errors with which the issue of the journal in which the translation appeared was flooded. It should have been printed *cases* of chronic hepatitis, not *cures.*

I shall be obliged to you if you refer to this correction in your next number.

Respectfully,

JOHN BARTLETT.

August 11, 1854.

